# The glutamatergic drive to breathe is reduced in severe but not moderate hypoxia in Damaraland mole-rats

**DOI:** 10.1242/jeb.246185

**Published:** 2023-10-06

**Authors:** Maiah E. M. Devereaux, Sarah Chiasson, Kate F. Brennan, Matthew E. Pamenter

**Affiliations:** ^1^ Department of Biology, University of Ottawa, Ottawa, ON K1N 6N5, Canada; ^2^ University of Ottawa Brain and Mind Research Institute, Ottawa, ON K1H 8M5, Canada

**Keywords:** AMPA receptor, NMDA receptor, Hypoxic ventilatory response, Hypoxic metabolic response

## Abstract

Damaraland mole-rats (*Fukomys damarensis*) are a hypoxia-tolerant fossorial species that exhibit a robust hypoxic metabolic response (HMR) and blunted hypoxic ventilatory response (HVR). Whereas the HVR of most adult mammals is mediated by increased excitatory glutamatergic signalling, naked mole-rats, which are closely related to Damaraland mole-rats, do not utilize this pathway. Given their phylogenetic relationship and similar lifestyles, we hypothesized that the signalling mechanisms underlying physiological responses to acute hypoxia in Damaraland mole-rats are like those of naked mole-rats. To test this, we used pharmacological antagonists of glutamatergic α-amino-3-hydroxy-5-methyl-4-isoxazolepropionic acid receptors (AMPARs) and *N*-methyl-d-aspartate receptors (NMDARs), combined with plethysmography, respirometry and thermal RFID chips, to non-invasively evaluate the role of excitatory AMPAR and NMDAR signalling in mediating ventilatory, metabolic and thermoregulatory responses, respectively, to 1 h of 5 or 7% O_2_. We found that AMPAR or NMDAR antagonism have minimal impacts on the HMR or hypoxia-mediated changes in thermoregulation. Conversely, the ‘blunted’ HVR of Damaraland mole-rats is reduced by either AMPAR or NMDAR antagonism such that the onset of the HVR occurs in less severe hypoxia. In more severe hypoxia, antagonists have no impact, suggesting that these receptors are already inhibited. Together, these findings indicate that the glutamatergic drive to breathe decreases in Damaraland mole-rats exposed to severe hypoxia. These findings differ from other adult mammals, in which the glutamatergic drive to breathe increases with hypoxia.

## Teaser

**Summary:** Hypoxia-tolerant species have reduced physiological responses to hypoxia. These reduced responses are partially controlled in Damaraland mole-rats.

## INTRODUCTION

Most adult terrestrial mammals are largely intolerant of low environmental oxygen (O_2_) tensions; however, a few species inhabit niches in which they encounter hypoxia on an intermittent or sustained basis (Bickler and Buck, 2007; Dzal et al., 2015). For example, inhabitants of subterranean environments often experience various degrees of hypoxia because of poor air circulation, limited diffusion of gases through the soil, and/or the metabolic consumption of O_2_ by densely grouped populations. Indeed, measurements from subterranean burrows of various species have reported ambient O_2_ concentrations ranging from 6 to 20.5% O_2_ (Holtze et al., 2018; Kennerly, 1964; Kuhnen, 1986; Maclean, 1981; Roper et al., 2001; Schaefer and Sadleir, 1979).

At the cellular level, hypoxia compromises aerobic energy production (Buck and Pamenter, 2006; Hochachka, 1986)*,* and exerts a strong evolutionary pressure on animals that dwell in such niches (Pamenter et al., 2020; Townley et al., 2022). In response, terrestrial mammals that inhabit hypoxic environments have evolved physiological and molecular adaptations that enable them to thrive with reduced O_2_ availability, primarily by increasing the supply of O_2_ to tissues and/or by decreasing demand for O_2_ (Boggs et al., 1984; Dzal et al., 2015). Oxygen supply can be increased by increasing ventilation or cardiac perfusion, improving blood O_2_ carrying capacity, or reducing diffusion barriers between blood vessels and tissues (Dzal et al., 2015). Conversely, reducing systemic metabolic demand can be achieved through a variety of strategies, including reductions in physical activity, thermogenesis, and various tissue and cellular functions, among others (Guppy and Withers, 1999; Hochachka, 1986; Hochachka et al., 1996; Pamenter and Powell, 2016; Teppema and Dahan, 2010). In general, most adult hypoxia-intolerant mammals rely primarily on increasing O_2_ supply (Frappell et al., 1992), even though this strategy is typically energetically demanding owing to the elevated metabolic effort associated with increased breathing or cardiac output. To increase O_2_ supply, most adult mammals increase ventilation (Frappell et al., 1992; Pamenter and Powell, 2016), which is referred to as the hypoxic ventilatory response (HVR). In contrast, hypoxia-tolerant adult mammals often exhibit robust metabolic rate suppression when they encounter a hypoxic environment [i.e. the hypoxic metabolic response (HMR)]. Importantly, some hypoxia-intolerant adult mammals with small body masses also reduce their metabolic demand in hypoxia. For example, mice exhibit robust metabolic rate suppression in hypoxia, whereas rats, star-nosed moles, ground squirrels and golden hamsters do not (Arias-Reyes et al., 2021; Devereaux et al., 2021; Dzal and Milsom, 2019; Wunnenberg et al., 1987). However, unlike in many hypoxia-tolerant species in which facultative reductions in metabolic rate are carefully regulated responses to reductions in O_2_ supply, the hypoxia-mediated suppression of metabolic rate in mice is associated with insufficient O_2_ delivery and is thus not an adaptive response.

The HVR is primarily mediated by signalling changes in the chemoreflex arc between peripheral carotid bodies and ventilatory control centres in the central nervous system (CNS). Broadly speaking, the HVR is predominately regulated by glutamatergic signalling in most hypoxia-intolerant adult mammals. Specifically, the onset of hypoxia is detected by peripheral arterial chemoreceptors of the carotid body, which signal to the medulla via afferent neurons in the carotid sinus nerve (Kumar and Prabhakar, 2012). Within the medulla, glutamate is released into the nucleus tractus solitarii (NTS), a region that, among other functions, controls ventilation (Housley and Sinclair, 1988; Yelmen, 2004). Because the release of glutamate into the NTS immediately precedes the HVR*,* it is postulated that glutamate is primarily responsible for the initial increase in ventilation (the first phase of the biphasic HVR). In support of this, injecting glutamate into the NTS is sufficient to initiate an HVR-like response in normoxia, whereas severing the carotid sinus nerve eliminates both glutamate release and the HVR (Mizusawa et al., 1994; Richter et al., 1999). Furthermore, both systemic injection as well as isolated NTS microinjection of *N*-methyl-d-aspartate receptor (NMDAR) antagonists abate, and in some cases eliminate, the HVR (Connelly et al., 1992; Mizusawa et al., 1994; Ohtake et al., 1998; Pamenter et al., 2014a,b).

In addition to NMDARs, glutamatergic α-amino-3-hydroxy-5-methyl-4-isoxazolepropionic acid receptors (AMPARs), also contribute to the HVR. In support of this, systemic intraperitoneal injection of AMPAR agonists increases phrenic nerve inspiratory bursts to respiratory muscle groups (ElMallah et al., 2015), and AMPA microinjection into the NTS results in a near HVR-like increase in respiratory frequency. Furthermore, broad-spectrum excitatory amino acid antagonists induce more wide-ranging inhibitory effects on hypoxic responses than NMDAR antagonism alone (Mizusawa et al., 1994; Pamenter et al., 2014b, 2015b), whereas the simultaneous injection of NMDAR and AMPAR antagonists into the NTS attenuates both cardiac and ventilatory responses to carotid body stimulation in rats (Braga et al., 2007; Haibara et al., 1999; Vardhan et al., 1993; Zhang and Mifflin, 1993). Taken together, these results indicate that glutamate released into the NTS in response to hypoxic stimulation of the carotid sinus nerve initiates the HVR via both NMDARs and AMPARs in most adult mammals. Conversely, few studies have explored the CNS control of metabolic responses to hypoxia in any adult species.

Unlike most adult mammals, many fossorial species, and especially social fossorial species, are hypoxia-tolerant and express a physiological phenotype to hypoxia that is divergent from that of adult hypoxia-intolerant mammals. For example, fossorial adult mammals typically reduce their metabolic rate in hypoxia (Arieli et al., 1977; Devereaux and Pamenter, 2020; Frappell et al., 1992; Guppy and Withers, 1999; Ivy et al., 2020; Tomasco et al., 2010), and, although it is not eliminated, the HVR of fossorial species tends to be smaller than that of surface-dwelling mammals and/or its onset occurs at a relatively lower level of environmental O_2_ than in non-fossorial mammals (i.e. their HVR is ‘blunted’). For example, the HVR threshold in fossorial species ranges from 5 to 12.5% O_2_ (Arieli and Ar, 1979; Barros et al., 2004; Boggs et al., 1998; Devereaux and Pamenter, 2020; Frappell et al., 1994; Ivy et al., 2020; Tomasco et al., 2010), whereas non-fossorial mammals hyperventilate closer to ∼16% O_2_ (Arieli and Ar, 1979; Hemingway and Nahas, 1952).

The CNS control of the HVR and HMR in fossorial species is poorly understood, but one such species where the CNS control of ventilation and metabolism has received some attention is the eusocial naked mole-rat. Naked mole-rats putatively experience deep but intermittent hypoxia in their crowded underground burrows (Buffenstein et al., 2022; Pamenter, 2022), and in a laboratory setting, can survive hypoxia down to 3% O_2_ for hours, and weeks in O_2_ levels of 8% (Chung et al., 2016; Farhat et al., 2020; Pamenter et al., 2019, 2018). In response to hypoxia, naked mole-rats reduce their metabolic rate (Pamenter et al., 2015a, 2019), partially through a rapid decrease in thermogenesis (Cheng et al., 2021; Kirby et al., 2018; Vandewint et al., 2019). Conversely, the ventilatory response of naked mole-rats to hypoxia is less well-defined owing to technical limitations of measuring ventilation in an animal with a body temperature (*T*_b_) that approaches ambient temperature (*T*_a_) in hypoxia (Cheng et al., 2021; Pamenter, 2023); however, it is generally accepted that they either decrease or maintain ventilation in hypoxia (Chung et al., 2016; Dzal et al., 2019; Pamenter et al., 2015a). Intriguingly, and unlike in all other adult mammals studied, modulation of glutamate receptors has no impact on the HVR (or the HMR) in naked mole-rats (Dzal et al., 2019).

Another fossorial species of interest is the Damaraland mole-rat (*Fukomys damarensis*), which is a close cousin of the naked mole-rat and is the only other eusocial mammal (Thomas et al., 2016). Much like other fossorial and hypoxia-tolerant species, Damaraland mole-rats have a blunted HVR with altered ventilation beginning in 12% O_2_, and a robust HMR, with metabolism decreasing by ∼56% in acute hypoxia (Ivy et al., 2020). Therefore, it is apparent that Damaraland mole-rats and naked mole-rats have a similar HVR and HMR, and thus Damaraland mole-rats may also have similar underlying control pathways. However, nothing is known about the CNS signalling mechanisms that control the HVR and HMR in Damaraland mole-rats.

To begin to address this knowledge gap, we examined the role of excitatory glutamatergic signalling in the HVR and HMR of Damaraland mole-rats. We hypothesized that the signalling pathways that regulate the HVR and HMR in Damaraland mole-rats are like those observed in naked mole-rats and do not rely upon excitatory glutamatergic signalling. To test this hypothesis, we measured ventilation, metabolism and thermoregulation in freely behaving Damaraland mole-rats exposed to normoxia (21% O_2_) and then acute hypoxia (1 h in 7 or 5% O_2_), with and without intraperitoneal injections of glutamatergic NMDAR or AMPAR antagonists.

**List of symbols and abbreviations**

ACR: air convection requirement
AMPAR: α-amino-3-hydroxy-5-methyl-4-isoxazolepropionic acid receptor
CNQX: cyanquixaline (6-cyano-7-nitroquinoxaline-2,3-dione)
CNS: central nervous system
*f*_R_: breathing frequency
HMR: hypoxic metabolic response
HVR: hypoxic ventilatory response
MK-801: dizocilpine
NMDAR: *N*-methyl-d-aspartate receptor
NTS: nucleus tractus solitarius
RER: respiratory exchange ratio
*T*_a_: ambient temperature
*T*_b_: body temperature
*V̇*_E_: minute ventilation
*V̇*_CO_2__: carbon dioxide production
*V̇*_O_2__: oxygen consumption rate
*V*_T_: tidal volume

## MATERIALS AND METHODS

### Animals

Damaraland mole-rats [*Fukomys damarensis* (Ogilby 1838)] were bred at the University of Ottawa group-housed in interconnected multi-cage systems at 22°C in 21% O_2_ and 0.04% carbon dioxide (CO_2_) and 50% humidity with a 12 h:12 h light:dark cycle. Animals were fed fresh tubers, vegetables, fruit and Pronutro cereal supplement *ad libitum*. The age of our experimental animals was selected based on a growth study of Damaraland mole-rats, which reported that this species reaches 90% of their full size by 600 days post-birth (Thorley and Clutton-Brock, 2019). Neural dimorphisms exist dependent on breeding status such that breeding animals have greater development in certain areas of the brain relative to subordinates of both sexes (Anyan et al., 2011), which may have unpredictable impacts on the neural control of physiological responses to hypoxia. As such, the queen and breeding male from our colony were omitted from this study.

### Experimental ethics and design

All experimental procedures were approved by the University of Ottawa's Animal Care Committee (protocol no. 2535) and conducted in accordance with the Animals for Research Act and other regulations of the Canadian Council on Animal Care. Experiments were performed during the daylight portion of the animals' daily light:dark cycle and the experimental time within this window was randomized for each individual animal and experimental protocol to remove any bias induced by circadian rhythms. Briefly, 10 adult subordinate Damaraland mole-rats (5 males, 5 females, age 1.5–3 years; 208.1±12.3 g, mean±s.e.m.) were subjected to each of six experimental conditions, consisting of exposure to 1 h of normoxia followed by intraperitoneal injections of: saline alone (controls), dizocilpine maleate (MK801, to block NMDARs; 2.5 mg kg^−1^; Reid and Powell, 2005; Wong et al., 1986) or cyanquixaline (CNQX, to block AMPARs; 5 mg kg^−1^; Dzal et al., 2019; Honore et al., 1988). Animals were then replaced in normoxic conditions for 1 h to assess the impact of the drug on the physiological parameters of interest, and were finally exposed to either 5 or 7% O_2_ for 1 h. The saline experiments in this study for both 7 and 5% O_2_ are also presented in Devereaux and Pamenter (2023). Animals were not fasted prior to experimental trials and all animals were exposed to all six experimental paradigms (3× injected treatments in two different hypoxic conditions) in random order. Animals were permitted a minimum of 1 week of rest between experiments to reduce any confounding effects of previous hypoxic exposures.

For each experiment, animals were individually placed unrestrained in a 1.0 l Plexiglass experimental chamber held at 22°C in normoxia (21% O_2_/0.04% CO_2_) until O_2_ consumption reached steady state for a minimum of 20 min. For the purposes of these experiments, we defined steady state as a period in which the concentration of O_2_ in the excurrent air did not fluctuate by more than 0.2% for a minimum of 15 min. Following this normoxic control period, animals were removed from the experimental chamber and injected intraperitoneally with either saline or one of the two drugs. Drugs were diluted to inject animals with a standard bolus of 2.5 µl g^−1^ of body mass and drug dosages were based on previous experiments performed on naked mole-rats in our lab, which were in turn based on studies in other small rodents (see references above). Dosages were updated for the Damaraland mole-rats on an as-needed basis. Animals must have been awake and not exhibiting signs of physical stress (e.g. haunching, scratching or licking injection site, irregular or strained breathing patterns, etc.) post-injection before they were subjected to experimentation.

After injection, animals were placed back into normoxia until O_2_ consumption reached steady state. This was done to control for any effects the drugs may have had on metabolic or ventilatory parameters while the animals were still breathing normoxic air, and to account for stress following the injection and related handling. Finally, O_2_ was lowered to 7% or 5%, taking up to 15–30 min to achieve the new hypoxic equilibrium in the experimental chamber and until metabolic parameters once again reached steady state. Our lab has previously conducted experiments in naked mole-rats using these same drug treatments in a 7% O_2_ hypoxic exposure. Therefore, we chose this O_2_ level for our experiments to permit simple comparison between the two species. However, in preliminary studies we did not observe a ventilatory response in 7% O_2_. Therefore, we repeated all experiments in a deeper level of hypoxia (5% O_2_) to better assess the effect of each drug on ventilation. Although Damaraland mole-rats have been studied in as low as 3% O_2_, pilot trials determined this was only tolerable for a maximum of 35 min with saline, and for less time in certain drug trials. Thus, 5% O_2_ was determined to be the lowest safe O_2_ level for the animals and we did not test deeper levels of hypoxia. Each experimental stage lasted approximately 1 h.

To maximize the sensitivity and resolution of metabolic rate measurements, gases were continuously supplied at 0.4 l min^−1^. As a result, animal-induced changes in the concentrations of O_2_ and CO_2_ would have altered the gas composition in the experimental chamber, reducing O_2_ by ∼0.4–2.0% and increasing CO_2_ by ∼0.1–1.0%, depending on their metabolic activity. However, standard practices in this field are based largely on studies in hypoxia-intolerant animals, such as mice, dogs and rats, which are considerably more sensitive to smaller changes in atmospheric O_2_ and CO_2_. Conversely, Damaraland mole-rats live at mild altitude in nature, in which atmospheric air is ∼18.5% O_2_, and their metabolic and ventilatory responses to hypoxia and hypercapnia are not activated until well beyond 19% O_2_ or 1% CO_2_ (Ivy et al., 2020; Zhang and Pamenter, 2019a,b). Therefore, it is unlikely that this impacted our evaluation of the regulation of the HVR and HMR in this species.

### Respirometry

During experimentation, the animal chamber was sealed and continuously ventilated with gas mixtures, set to the desired fractional gas composition by calibrated rotameters (Praxair, Mississauga, ON, Canada). Incurrent air was bubbled in distilled H_2_O prior to entering the animal chamber. Excurrent air passed through an RH-300 Water Vapour Pressure Analyzer to measure humidity, and the average incurrent gas humidity was 94.5±3.1%, which is consistent with previous experiments using similar equipment in our laboratory (Clayson et al., 2020). Excurrent gas passed through a desiccant medium before entering the O_2_ and CO_2_ analyzers. The final 10-min period of stable activity from each experimental stage was used for data analysis. To determine metabolic rate, eqns 10.6 and 10.7 from Lighton (2008) were used to calculate the rate of O_2_ consumption (*V̇*_O_2__, ml min^−1^ kg^−1^) and the rate of CO_2_ production (*V̇*_CO_2__, ml min^−1^ kg^−1^), respectively:
(1)

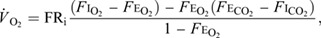

(2)

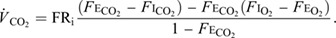


In these equations, FR_i_ is the incurrent flow rate (ml min^−1^), *F*i_O_2__ and *F*i_CO_2__ are the fractional concentrations of incurrent O_2_ and CO_2_ of dry gas, respectively, and *F*e_O_2__ and *F*e_CO_2__ are the fractional concentrations of excurrent O_2_ and CO_2_ from the experimental chamber, respectively (Lighton, 2008). Respiratory exchange ratios (RERs) were calculated as *V̇*_CO_2__/*V̇*_O_2__.

### Thermal measurements

Body temperature (*T*_b_, °C) was recorded non-invasively every 10 min throughout all experiments using an RFID microchip reader (Allflex USA Inc., Dallas, TX, USA) to scan previously implanted subcutaneous RFID microchips (Destron Fearing) along the back flank of the animal. Chamber temperature was recorded at each inflow period using a custom thermocouple.

### Whole-body plethysmography

Attached to the experimental chamber was an identical second chamber that acted as a reference chamber. Continuous monitoring by a differential pressure transducer connected between the two chambers amplified small pressure fluctuations in the experimental chamber, allowing us to detect and measure breaths at 1000 Hz. Before each trial, the transducer was calibrated by injecting six known volumes of air (0.1, 0.2, 0.3, 0.4, 0.5 and 0.6 ml) 10 times into the experimental chamber. Injections were performed with continuous airflow through the pressure-sealed system at the same respiratory frequency (*f*_R_, breaths min^−1^) the animal was observed to have under normoxic/normocapnic conditions. To calculate tidal volume (*V*_T_, ml kg^−1^) and *f*_R_, five breath sets consisting of a minimum of 10 consecutive and clearly defined breaths within the same 10 min period as that used for metabolic rate calculations were analyzed. The Drorbaugh and Fenn (1955) equation was used to calculate *V*_T_:
(3)

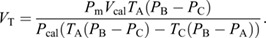


The average oscillation height was taken from each breath set, representing the average total pressure deflection of a breath (*P*_m_). *P*_cal_ (V) and *V*_cal_ (µl) are the pressure deflection and volume of a known calibrated volume, respectively. The average oscillation height of each calibration set was plotted against its respective volume and used to create a linear relationship. The point on this line representing 0.2 ml was chosen as *P*_cal_ and *V*_cal_. *T*_A_ is the body temperature of the animal (in K) and *T*_C_ is the temperature of the chamber (K), both recorded at the end of the 10 min period. *P*_B_ is the barometric pressure in the lab (mmHg) as measured by the O_2_ analyzer. *P*_A_ is the vapour pressure of water at the animal's body temperature (mmHg) and *P*_C_ is the partial pressure of water vapour (mmHg) in the incurrent gas stream (Drorbaugh and Fenn, 1955). *P*_A_ was calculated using relative humidity (%) of excurrent air, animal temperature (°C) and barometric pressure (kPa). *P*_C_ used relative humidity (%) of incurrent air, chamber temperature (°C) and barometric pressure (kPa). The same breath samples were used to calculate *f*_R_. Minute ventilation (*V̇*_E_) was calculated as the product of *f*_R_ and *V*_T_. The air convection requirements (ACRs) for O_2_ and CO_2_ were calculated as the quotient of *V̇*_E_ and *V̇*_O_2__ or *V̇*_CO_2__, respectively.

### Analysis

Ventilatory and metabolic data were collected using LabChart software (ADInstruments, Colorado Springs, CO, USA) and analyzed in PowerLab (ADInstruments). Statistical analysis was performed using commercial software (Prism v.9.2.0, GraphPad Software Inc., La Jolla, CA, USA). Responses to hypoxia and changes mediated by MK801 or CNQX administration were not different between sexes and so these data were pooled in our analysis. *P*<0.05 was considered the threshold for statistical significance. Data are presented in box and whisker plots. Statistical significance was evaluated using a two-way repeated-measures analysis of variance (RM-ANOVA) or mixed-effects model, as appropriate, to test for interactions between normoxia and hypoxia (7% or 5% O_2_), or drug treatments. Tukey's or Šidák's multiple comparisons tests were performed on each dependent variable to determine significance.

## RESULTS

### MK801 reduces *V̇*_O_2__ in severe hypoxia

**Fig. 1.**
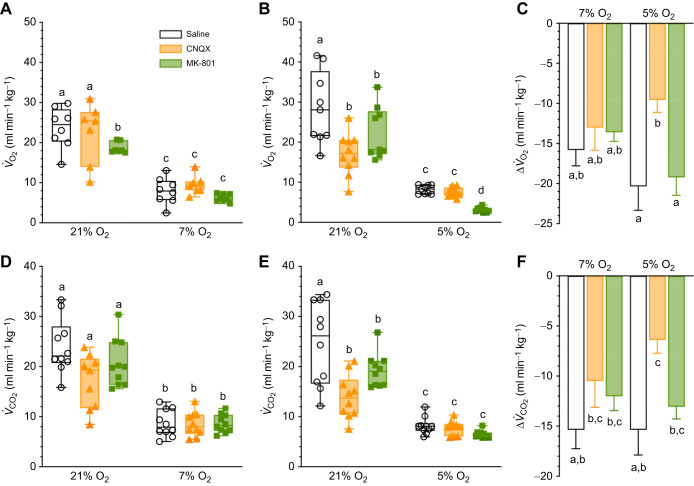
**Glutamatergic receptor antagonism enhances the hypoxic metabolic response in severe hypoxia.** (A,B,D,E) Summaries of O_2_ consumption rate (*V̇*_O_2__; A,B) and CO_2_ production rate (*V̇*_CO_2__; D,E) from Damaraland mole-rats exposed to 21% O_2_, before and after intraperitoneal injections of saline alone, the α-amino-3-hydroxy-5-methyl-4-isoxazolepropionic acid receptor (AMPAR) antagonist 6-cyano-7-nitroquinoxaline-2,3-dione (CNQX; 5 mg kg^−1^; orange bars and symbols), or the *N*-methyl-d-aspartate receptor (NMDAR) antagonist dizocilpine (MK-801; 2.5 mg kg^−1^; green bars and symbols), dissolved in saline, and subsequent exposure to acute hypoxia (7 or 5% O_2_; A,D and B,E, respectively). Summary data are presented as box and whisker plots of min–max with individual replicates shown from *n=*7–10 biological replicates per dataset. (C,F) Summaries of the magnitude of the hypoxic metabolic response from animals treated as in A,B and D,E. Data are presented as means±s.e.m. Different letters indicate significance as indicated using a two-way ANOVA or mixed-effects model with Tukey's *post hoc* tests, *P<*0.05.

First, we evaluated the impact of glutamatergic receptor antagonism in normoxia and on the HMR. In saline controls, metabolic variables decreased significantly with the onset of hypoxia. Specifically, both *V̇*_O_2__ and *V̇*_CO_2__ decreased by ∼65–75% in both levels of hypoxia (*F*_1,25_=153.5, *P<*0.0001, *F*_1,19_=144.3, *P<*0.0001, *F*_1,27_=139.9, *P<*0.0001, and *F*_1,26_=149.0, *P<*0.0001 for *V̇*_O_2__ in 7 and 5% O_2_ and *V̇*_CO_2__ in 7 and 5% O_2_, respectively; Fig. 1). The magnitude of the HMR in saline-treated animals was not different between hypoxia levels for either *V̇*_O_2__ (*P*=0.6914; Fig. 1C) or *V̇*_CO_2__ (*P*>0.999; Fig. 1F).

In drug-treated animals, CNQX tended to reduce *V̇*_O_2__ and *V̇*_CO_2__ in normoxia, although this effect was not significant in all treatment groups (*P*<0.0001 for both in 5% O_2_ experiments; Fig. 1). With the onset of hypoxia, metabolic variables decreased by >50% (*P*<0.0001 and *P*=0.0006 for *V̇*_O_2__ in 7 and 5% O_2_, respectively; *P*=0.0001 and 0.003 for *V̇*_CO_2_ _in 7 and 5% O_2_, respectively), and to levels that were similar to saline-treated animals in the same degree of hypoxia (*P*>0.9999 for all comparisons). Relative to saline controls, the magnitude of the HMR in CNQX-treated animals was reduced in the 5% but not the 7% O_2_ treatment groups (*P*=0.0079 for *V̇*_O_2__ and 0.0215 for *V̇*_CO_2__; Fig. 1C,F). This reduction was due to the lower metabolic rate in animals treated with CNQX in normoxia and not to a reduction in the absolute metabolic rate in hypoxia.

Similarly, following MK801 injection, *V̇*_O_2__ and *V̇*_CO_2__ were both reduced by ∼20% in normoxia (*P*<0.0001 for both; Fig. 1). With the onset of hypoxia, *V̇*_O_2__ decreased ∼65–85% further (*P*<0.0001 for all). This hypoxic *V̇*_O_2__ level was similar to that of saline controls in 7% O_2_ (*P*=0.8035) but was reduced relative to controls in 5% O_2_ (*P*=0.0456). Similarly, *V̇*_CO_2__ decreased by ∼60–70% in hypoxia in CNQX-treated animals (*P*<0.0001 for both), and the final level of *V̇*_CO_2__ was similar to that of saline controls in these animals in both 7 and 5% O_2_ (*P*=0.8035 and 0.7213, respectively). Relative to saline controls, the magnitude of the HMR was not affected by MK801 (Fig. 1C,D).

### Damaraland mole-rats switch from mixed lipid/carbohydrate metabolism to entirely carbohydrate metabolism in hypoxia

**Fig. 2.**
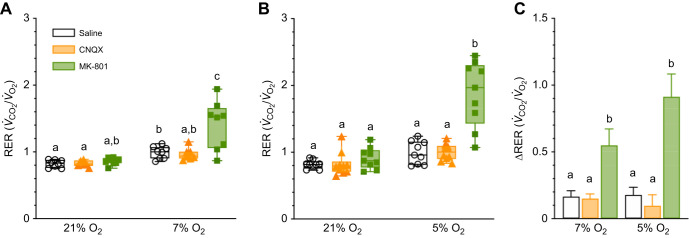
**Damaraland mole-rats switch from mixed carbohydrate/lipid metabolism in normoxia to entirely carbohydrate metabolism in hypoxia.** (A,B) Summaries of respiratory exchange ratios (RERs) from Damaraland mole-rats exposed to 21% O_2_, before and after intraperitoneal injections of saline, CNQX (5 mg kg^−1^; orange bars and symbols) or MK-801 (2.5 mg kg^−1^; green bars and symbols), dissolved in saline, and subsequent exposure to acute hypoxia (7 or 5% O_2_; A,D and B,E, respectively). Summary data are presented as box and whisker plots of min–max with individual replicates shown from *n*=7–10 biological replicates per dataset. (C) Summary of the magnitude of change in these variables from animals treated as in A and B. Data are presented as means±s.e.m. Different letters indicate significance as indicated using a two-way ANOVA or mixed-effects model with Tukey's *post hoc* tests, *P<*0.05. Abbreviations are as described in the legend of Fig. 1.

Comparing the rates of O_2_ consumption and CO_2_ production (i.e. the RER) provides an indirect measure of metabolic fuel usage (Lau et al., 2017; Simonson and DeFronzo, 1990). Hypoxic treatment had a significant impact on the RER in both experimental groups (*F*_1,19_=35.13, *P*<0.0001 and *F*_1,25_=48.10, *P*<0.0001 in 7 and 5% O_2_, respectively; Fig. 2A,B). In saline-treated animals, the RER changed from 0.82 in normoxia to 0.990 in both 7 and 5% O_2_. Antagonism of AMPARs had no effect on the RER in normoxia or hypoxia (*P*>0.9999 for all comparisons). With the onset of hypoxia, the RER increased in CNQX-treated animals to 0.97 and 1.00 in 7 and 5%, respectively, which was not different from saline controls in hypoxia (*P*=0.9944 and 0.9996 in 7 and 5%, respectively). MK801 treatment also had no effect on the RER in normoxia (*P*=0.9668 and 0.7855 in 7 and 5% O_2_ experiments, respectively), but the RER in these animals increased significantly to 1.41 and 1.88 in 7 and 5% hypoxia, respectively (*P*<0.0001 for both). As a result, the magnitude of change in the RER was greater with MK801 than in other treatment groups (*P*<0.0001 for all comparisons; Fig. 2C).

### Thermoregulation is decreased in hypoxia, independent of AMPAR or NMDAR regulation

**Fig. 3.**
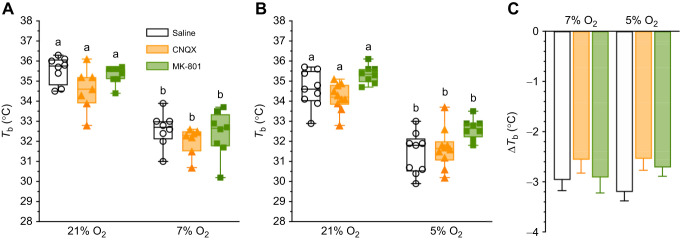
**Body temperature (*T*_b_) is decreased in hypoxia independent of glutamate receptor antagonism.** (A,B) Summaries of *T*_b_ from Damaraland mole-rats exposed to 21% O_2_, before and after intraperitoneal injections of saline, CNQX (5 mg kg^−1^; orange bars and symbols) or MK-801 (2.5 mg kg^−1^; green bars and symbols), dissolved in saline, and subsequent exposure to acute hypoxia (7 or 5% O_2_; A and B, respectively). Summary data are presented as box and whisker plots of min–max with individual replicates shown from *n*=7–10 biological replicates per dataset. (C) Summary of the magnitude of change of *T*_b_ from animals treated as in A and B. Different letters indicate significance as indicated using a two-way ANOVA or mixed-effects model with Tukey's *post hoc* tests, *P<*0.05. Abbreviations are as described in the legend of Fig. 1.

We also measured *T*_b_ to gain insight into the impact of hypoxia on thermoregulation because many small mammals reduce *T*_b_ to facilitate metabolic savings in hypoxia (Barros et al., 2001; Wood and Gonzales, 1996; Wood and Stabenau, 1998). We found that *T*_b_ decreased by ∼3°C in saline-treated animals exposed to both 7 and 5% O_2_ (*F*_1,20_=90.49, *P*<0.0001 and *F*_1,24_=584.2, *P*<0.0001 for 7 and 5% O_2_, respectively; Fig. 3A). In drug trials, CNQX and MK801 had no significant effect on *T*_b_ in normoxia, or on the hypoxic change in *T*_b_ (*P*>0.9999 for all comparisons; Fig. 3A,B). Similarly, the magnitude of the *T*_b_ change was similar across all treatment conditions and levels of hypoxia (*F*_1,__44_=0.001274, *P*=0.9717; Fig. 3C).

### Ventilation is decreased in severe but not moderate hypoxia

**Fig. 4.**
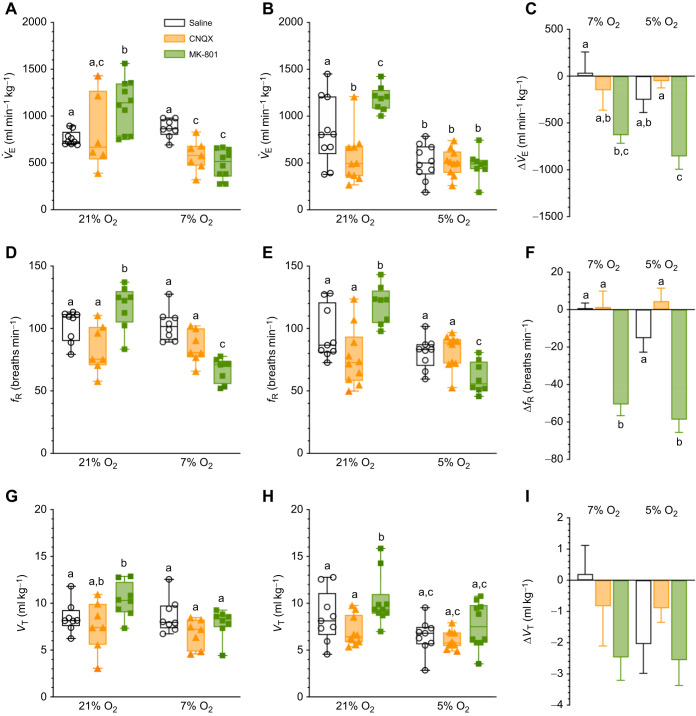
**The glutamatergic drive to breathe decreases with severe hypoxia.** (A,B,D,E,G,H) Summaries of minute ventilation (*V̇*_E_; A,B), breathing frequency (*f*_R_; D,E) and tidal volume (*V*_T_; G,H) from Damaraland mole-rats exposed to 21% O_2_, before and after intraperitoneal injections of saline, CNQX (5 mg kg^−1^; orange bars and symbols) or MK-801 (2.5 mg kg^−1^; green bars and symbols), dissolved in saline, and subsequent exposure to acute hypoxia (7 or 5% O_2_; A,D,G and B,E,H, respectively). Summary data are presented as box and whisker plots of min–max with individual replicates shown from *n=*7–10 biological replicates per dataset. (C,F,I) Summaries of the magnitude of change in these variables from animals treated as in A,B, D,E and G,H. Data are presented as means±s.e.m. Different letters indicate significance as indicated using a two-way ANOVA or mixed-effects model with Tukey's *post hoc* tests, *P<*0.05. Abbreviations are as described in the legend of Fig. 1.

Next, we measured changes in ventilation and its component parameters (*f*_R_ and *V*_T_) to evaluate the HVR. Relative to normoxia, saline-treated animals exhibited a 40% reduction in *V̇*_E_ in 5% O_2_ (*F*_1,25_=53.31, *P*<0.0001; Fig. 4A) but no change in 7% O_2_ (*P*=0.6807; Fig. 4B). However, although the change in 5% O_2_ was significant when considering the averaged data, the magnitude of change was not significant when comparing across datasets (*F*_1,48_=1.249, *P*=0.2693; Fig. 4C). The change in *V̇*_E_ in 5% O_2_ was mediated by a non-significant ∼15% reduction in *f*_R_ (Fig. 4D; *P*=0.1243) and a significant 25% reduction in *V*_T_ (*F*_1,26_=17.76, *P*=0.0003; Fig. 4H). Conversely, both *f*_R_ and *V*_T_ were unchanged in 7% O_2_ (*P>*0.9999 for all comparisons; Fig. 4G,H), and the magnitude of change was not different between control datasets (*F*_1,44_=1.596, *P*=0.2131; Fig. 4F,I).

### AMPAR or NMDAR antagonism reduces ventilation in moderate hypoxia

AMPAR antagonism did not impact *V̇*_E_ in normoxia in the 7% O_2_ experiments (*P*=0.9929; Fig. 4A) but caused an ∼35% reduction in *V̇*_E_ in normoxia in the 5% O_2_ experiments (*P*=0.0219; Fig. 4B). No further changes were observed in these animals with subsequent hypoxia exposure in either experimental group (*P*=0.1226 and 0.9009 for 5 and 7% O_2_, respectively). However, it is notable that *V̇*_E_ was ∼34% lower in CNQX-treated animals in 7% O_2_ than in saline-treated animals in the same conditions (*P*=0.0073), but was not different from saline-only controls in 5% O_2_ (*P*=0.9985). The magnitude of the HVR in CNQX-treated animals was not different from that of saline controls (Fig. 4C). The CNQX-mediated changes in *V̇*_E_ in normoxia resulted from non-significant reductions in *f*_R_ and *V*_T_, which each decreased by ∼11–20% with drug injections (*P*=0.572 and 0.0692 for *f*_R_ in 7 and 5% O_2_, respectively, and 0.7362 and 0.3332 for *V*_T_ in 7 and 5% O_2_, respectively; Fig. 4D–I). Neither variable changed with hypoxic exposure and the magnitude of change was not significant for *f*_R_ or *V*_T_ with CNQX treatment in either hypoxia level (Fig. 4F,I)

Whereas CNQX treatment tended to slightly decrease ventilatory parameters in normoxia, MK801 tended to increase normoxic ventilation. Specifically, MK801-treatment increased *V̇*_E_ by ∼40–50% in all experimental protocols (*P*=0.0022 and 0.0073 for 7 and 5% O_2_ trials, respectively; Fig. 4A,B). These changes were mediated by 15–20% increases in both *f*_R_ and *V*_T_ (*P*=0.0472, 0.0492, 0.0114 and 0.0074 for *f*_R_ and *V*_T_ in 7 and 5% O_2_, respectively; Fig. 4D,E,G,H). With the onset of hypoxia, V̇_E_ in MK801-treated animals decreased to a similar value as in saline-treated controls in both the 5 and 7% O_2_ experimental groups, mediated by offsetting and minor decreases in *f*_R_ (*P*<0.0001 for both; Fig. 4D,E) and increases in *V*_T_ (*P*<0.0055 and 0.0052, respectively; Fig. 4G,H). Relative to saline-treated controls in the same hypoxic conditions, *f*_R_ was reduced by 25–35% (*P*=0.0225 and <0.0001 for 7 and 5% O_2_, respectively), but *V*_T_ was unchanged (*P*>0.9999 for all comparisons). As a result of these changes, the magnitude of the decrease of *V̇*_E_ and *f*_R_ was markedly enhanced in both 7 and 5% O_2_ with MK801 treatment (*F*_2,48_=11.75, *P*<0.0001 for *V̇*_E_, and *F*_2,44_=40.88, *P*<0.0001 for *f*_R_; Fig. 4C,F), but not *V*_T_ (*F*_2,46_=2.456, *P*=0.0969; Fig. 4I).

### Glutamate receptor antagonism decreases the ACR in moderate hypoxia but increases it in severe hypoxia

**Fig. 5.**
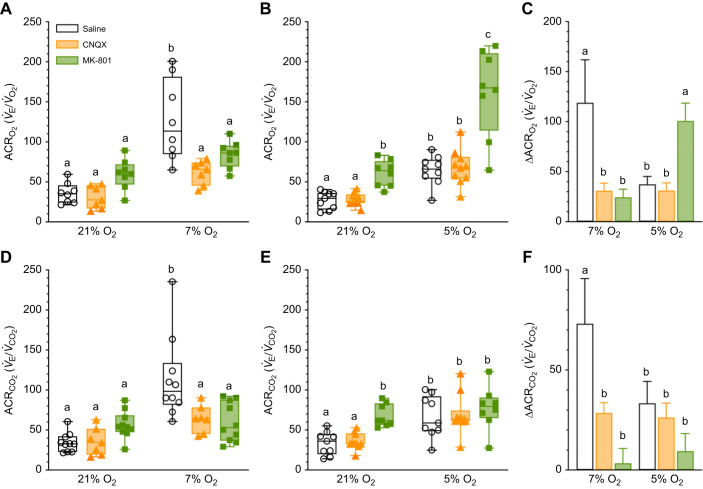
**Glutamatergic receptor antagonism attenuates hyperventilation in moderate but not severe hypoxia.** (A,B,D,E) Summaries of the air convection requirements of O_2_ (ACR_O_2__; A,B) and CO_2_ (ACR_CO_2__; D,E) from Damaraland mole-rats exposed to 21% O_2_, before and after intraperitoneal injections of saline, CNQX (5 mg kg^−1^) or MK-801 (2.5 mg kg^−1^; green bars and symbols), dissolved in saline, and subsequent exposure to acute hypoxia (7 or 5% O_2_; A,D and B,E, respectively). Summary data are presented as box and whisker plots of min–max with individual replicates shown from *n=*7–10 biological replicates per dataset. (C,F) Summaries of the magnitude of change in these variables from animals treated as in A,B and D,E. Different letters indicate significance as indicated using a two-way ANOVA or mixed-effects model with Tukey's *post hoc* tests, *P<*0.05. Abbreviations are as described in the legend of Fig. 1.

ACRs are the ultimate indicator of hyperventilation as they provide a combined measure of ventilation relative to metabolic demand. Therefore, we next calculated ACRs to determine whether Damaraland mole-rats hyperventilate in hypoxia. Interestingly, we found that in saline-treated controls, the ACR_O_2__ and ACR_CO_2__ increased∼3.5- to 4-fold in 7% O_2_ but only ∼2- to 2.5-fold in 5% O_2_ (*F*_2,47_=26.54, *P*<0.0001 for O_2_, *F*_2,51_=2.858, *P*<0.0001 for CO_2_; Fig. 5A,B,D,E). As a result, the magnitude of change in the ACR was 2-fold larger in 7% versus 5% O_2_ (Fig. 5C).

CNQX injection had no effect on the ACRs in normoxia or severe hypoxia (*P*>0.9999 for all comparisons) but reduced the ACR by half in 7% O_2_ (*P*<0.0001 for both ACR_O_2__ and ACR_CO_2__ versus saline controls; Fig. 5A,D). Furthermore, CNQX reduced the change in the magnitude of the ACRs by ∼60–75% in the 7% O_2_ group (*P*=0.0421 and 0.0185 for ACR_O_2__ and ACR_CO_2__, respectively; Fig. 5C,F), but not the 5% O_2_ group (*P*>0.9999 for all comparisons).

Conversely, MK801 tended to increase the ACRs in normoxia, although this trend only reached significance in the 5% O_2_ trials (*P*=0.1102 and 0.1786 for the ACR_O_2__ and ACR_CO_2__ in 7% O_2_, respectively, and 0.0280 and 0.0032 for the ACR_O_2__ and ACR_CO_2__ in 5% O_2_, respectively). In hypoxia, MK801 reduced the ACR by 33–50% in 7% O_2_ (*P*<0.0001 for both ACR_O_2__ and ACR_CO_2__ versus saline controls; Fig. 5A,C), but increased the ACR_O_2__ by ∼3-fold over similarly treated saline controls in 5% O_2_ (*P<*0.0001; Fig. 5B).

## DISCUSSION

In the present study, we evaluated a role for excitatory glutamatergic signalling in regulating the hypoxic ventilatory, metabolic and thermoregulatory responses of fossorial Damaraland mole-rats to acute hypoxia. Our study yielded several important findings. First, glutamate receptor antagonism has mixed but generally mild impacts on metabolic rate and ventilation in normoxia that tend to be receptor-type specific. Second, Damaraland mole-rats have a robust HMR, which is not sensitive to AMPAR antagonism and is slightly enhanced by NMDAR antagonism. Third, ventilation decreases in severe but not moderate hypoxia, but ventilation in moderate hypoxia is reduced with glutamatergic receptor antagonism. Therefore, the HVR manifests in less severe hypoxia when these receptors are blocked. Taken together, these findings indicate that Damaraland mole-rats have an endogenous glutamatergic drive to breathe and that this drive is switched off with more severe hypoxia. Specifically, both AMPARs and NMDARs contribute to this ventilatory drive to breathe in 7% O_2_ but not in 5% O_2_. These findings are contrary to those from previous studies in closely related naked mole-rats, in which excitatory glutamatergic signalling did not impact the HVR, but also differ from results in other adult mammals in which upregulated glutamatergic signalling was central to the HVR. Indeed, and to our knowledge, downregulation of glutamatergic regulation of breathing during acute hypoxia has not been shown previously in any species whose normoxic ventilation is primarily driven by glutamatergic signalling.

### Damaraland mole-rats rely primarily on a robust HMR, mediated in part by decreased thermogenesis, and have a blunted HVR

We report that Damaraland mole-rats rely primarily on a robust HMR in acute hypoxia, which is partially mediated by reduced thermoregulation. In addition, hypoxia-mediated changes in breathing are blunted in Damaraland mole-rats and do not manifest until 5% O_2_ under our study conditions. These findings are generally consistent with those in numerous other fossorial small mammal species (Arieli and Ar, 1979; Arieli et al., 1977; Barros et al., 2004; Boggs et al., 1998; Frappell et al., 1992, 1994; Guppy and Withers, 1999; Tomasco et al., 2010), including numerous other African mole-rats (Devereaux and Pamenter, 2020; Ivy et al., 2020).

Two previous studies have explored metabolic, ventilatory and thermoregulatory responses to acute hypoxia in Damaraland mole-rats (Ivy et al., 2020; Zhang and Pamenter, 2019b). These studies presented somewhat conflicted data regarding these physiological responses. Specifically, Zhang and Pamenter (2019b) did not observe an HMR in acute hypoxia, whereas Ivy et al. (2020) and the present study report robust depressions in metabolic rate in hypoxia. However, all three studies consistently report decreasing *T*_b_ with progressive hypoxia. In contrast, Zhang and Pamenter (2019b) and Ivy et al. (2020) reported a relatively high RER in normoxia that did not change in hypoxia, whereas we report an increase in RER with hypoxia, indicating a switch from mixed lipid/carbohydrate metabolism to complete reliance on carbohydrates in hypoxia. Regarding breathing, both Zhang and Pamenter (2019b) and Ivy et al. (2020) reported increases in *V̇*_E_ with progressive hypoxia, whereas we report an HVR that consists of a decrease in *V̇*_E_.

Importantly, however, these studies used somewhat divergent protocols and populations of animals. Specifically, Ivy et al. (2020) and Zhang and Pamenter (2019b) used progressive hypoxia protocols in which animals were ‘stepped down’ from moderate to more severe hypoxia for 30 or 60 min at each level, respectively. Conversely, we used an acute change to a single and relatively severe level of hypoxia in each experiment. A more gradual onset of hypoxia may impact the recruitment of metabolic and ventilatory adaptations to hypoxia. Furthermore, Ivy et al. (2020) used wild-caught animals that were likely juveniles, given their average mass of ∼125 g. Conversely, Zhang and Pamenter (2019b) and the present study used adult animals that were lab-raised and with average masses of ∼210–230 g. In general, juvenile and adult animals have somewhat different physiological responses to hypoxia in most mammals (Frappell et al., 1992; Mortola, 1999, 2004), and this may explain some of the differences between these studies.

Perhaps most important is the difference in study temperature between these three reports. Ivy et al. (2020) conducted experiments in a warm laboratory with an ambient temperature of 28°C. Similarly, Zhang and Pamenter (2019b) conducted experiments in the thermoneutral zone of Damaraland mole-rats: 30°C. Conversely, the present study was conducted at a more typical room temperature of 22°C. This relatively cooler temperature would increase the scope of metabolic savings gained from reduced thermogenesis and may have affected the need to recruit ventilatory responses in acute hypoxia. Further studies are warranted to carefully explore physiological responses to hypoxia throughout development in this species and in different temperatures to resolve these differences.

### The glutamatergic drive to breathe decreases in severe hypoxia

We report that both AMPAR and NMDAR inhibition reduces the ventilatory drive to breathe in 7% but not 5% O_2_. Numerous studies have explored the role of glutamatergic receptors in mediating the HVR (see Introduction). With overwhelming consistency, these studies report that the HVR manifests as an increase in ventilation with acute hypoxia in adult mammals, and that antagonism of glutamate receptors does not impact ventilation in normoxia but reduces or abolishes the HVR. Thus, in most adult mammals there is an increased drive to breathe in hypoxia that is mediated by upregulated glutamatergic signalling. In Damaraland mole-rats, the direction of change in absolute ventilation mediated by glutamate receptor antagonism is like that in other adult mammals (i.e. a net decrease in total ventilation), indicating that glutamatergic signalling is excitatory in this system. However, unlike in other adult mammals, there does not appear to be an upregulation of glutamatergic signalling in the hypoxic Damaraland mole-rat ventilatory control reflex arc. Instead, the glutamatergic drive to breathe may decrease with severe hypoxia, as suggested by our observation that glutamate receptor antagonism reduces ventilation in 7% but not 5% O_2_. Importantly, because the HVR in Damaraland mole-rats is a decrease in ventilation as opposed to the increase reported in all other adult mammals (except naked mole-rats), the impact of glutamate receptor antagonism is to further reduce ventilation, and also to induce the HVR in less severe levels of hypoxia and thereby reduce the ‘blunting’ of the HVR in this species.

### Study limitations

Both CNQX and MK801 impacted breathing and metabolic rate; therefore, these drugs and the concentrations employed are efficacious in our study. Nonetheless, drugs were injected intraperitoneally and thus the specificity of their action is unclear. Unfortunately, cranial maps are not available for Damaraland mole-rats, and so stereotaxic implantation of permanent cannulas that target the respiratory brainstem is not yet feasible in this species. However, intraperitoneal injections of these pharmacological agents have been employed successfully in numerous studies in various small rodent species (e.g. Berrino et al., 2003; Dzal et al., 2019; Jeon et al., 2017; McGuire et al., 2008; Mead and Stephens, 1999; Murschall and Hauber, 2005; Reid and Powell, 2005; Velisek et al., 1995; Xiang et al., 2018), indicating that these agents cross the blood–brain barrier to interact with AMPARs and NMDARs, which are not found outside of the CNS.

### Conclusions

In the present study, we demonstrated that AMPA and NMDA receptors partially mediate metabolic rate and ventilation in Damaraland mole-rats exposed to either normoxia or acute hypoxia. However, unlike in most other adult mammals, in which glutamatergic signalling is upregulated in hypoxia, leading to a net increase in ventilation, AMPAergic and NMDAergic signalling became downregulated with severe hypoxia in Damaraland mole-rats, contributing to a net decrease in ventilation. Conversely, we report only minor effects of NMDAR inhibition and no effect of AMPAR inhibition on the HMR and, unfortunately, we are not aware of any studies in other species in which the role of glutamatergic receptors in mediating the HMR has been explored. Further study is clearly required to fully understand how the HMR and HVR are regulated in this species. Exploring the role of inhibitory synaptic pathways may be relevant given the net decrease in both metabolism and ventilation during acute hypoxia in this species.
